# Preparing towards Preventing and Containing an Ebola Virus Disease Outbreak: What Socio-cultural Practices May Affect Containment Efforts in Ghana?

**DOI:** 10.1371/journal.pntd.0004852

**Published:** 2016-07-18

**Authors:** Philip Baba Adongo, Philip Teg-Nefaah Tabong, Emmanuel Asampong, Joana Ansong, Magda Robalo, Richard M. Adanu

**Affiliations:** 1 Department of Social and Behavioural Sciences, School of Public Health, University of Ghana, Legon, Accra, Ghana; 2 World Health Organization Country Office for Ghana, Accra, Ghana; 3 Department of Population, Family and Reproductive Health, School of Public Health, University of Ghana, Legon, Accra, Ghana; Liverpool School of Tropical Medicine, UNITED KINGDOM

## Abstract

**Background:**

Ebola Virus Disease (EVD) is a condition with high fatality. Though the disease is deadly, taking precautions to reduce contact with infected people and their secretions can prevent cross- infection. In the 2014 EVD outbreak, socio-cultural factors were identified to be responsible for the spread of the disease in the three most affected countries in West Africa. In this light, we undertook this study to identify socio-cultural factors that may influence the prevention and containment of EVD in Ghana and ways to address such practices.

**Methods:**

We conducted a descriptive qualitative study in five regions in Ghana. Twenty-five focus group discussions (5 in each region) with community members (4 in each region) and nurses (1 in each region) were conducted. In addition, forty (40) in-depth interviews were conducted with various stakeholders and opinion leaders; eight in each region. All interviews were recorded using a digital voice recorder and transcribed. With the aid of Nvivo 10 for windows, we analyzed the data using framework analysis.

**Results:**

We found that socio-cultural practices, such as care of the body of dead and burial practices, widowhood rites and anointing children with water used to rinse the dead, were common. These practices require individuals coming into direct contact with either the dead or items used to take care of the dead. Social norms also require frequent handshakes in all social gatherings such as funeral, and religious congregations. We also found that self-medication (using herbs and orthodox medications) was a common practice. People use both biomedical and non-orthodox health outlets either simultaneously or in sequence in times of ill-health.

**Conclusion:**

The study concludes that high risk socio-cultural practices were common among Ghanaians and generally perceived as indispensable. These high risk practices may hinder containment efforts in the event of an outbreak. Community leaders should be engaged in any social mobilization to modify these practices as part of preparation efforts.

## Author Summary

The 2014 Ebola Virus Disease outbreak emerged as the most devastating outbreaks in recent history. This outbreak spread across continents with West Africa remaining the epicenter. Although, the three most affected countries have been declared Ebola free, the recent re-emergence of cases in Sierra Leone require that countries in West Africa do not relent in their EVD containment efforts. Burial and funeral practices are high-risk practices that can facilitate the spread of ebola and hinder containment efforts. These practices vary across countries and regions. In this study, we found that despite the social and contextual difference across regions in Ghana, common high-risk practices exist and perceived to be indispensable. We also found that little attention were given to these practices in risk communication and community engagements. These practices required either touching the dead or items used in taking care of the dead. Widowhood rites require a widow drinking water used to bath the deceased husband to show her innocence in the husband’s death. Findings of this study attest to the need for improved dialogue with community leaders to provide alternatives to these high-risk socio-cultural practices to be able to contain an Ebola outbreak. Countries with similar socio-cultural practices could also use the findings of this study to guide behavioural change communication strategies.

## Introduction

Ebola Virus Disease (EVD) is a condition with high infectivity and case fatality rate. The case fatality rate of the 2014 EVD epidemic was initially estimated as 70% at the beginning of the epidemic [1,2]. However, the case fatality declined with the establishment of treatment centres and provision of logistics to care for infected individuals. This epidemic which started in a rural community in Guinea was declared a public health emergency of international concern by the World Health Organization as the outbreak spread across countries. Statistics show that the number of confirmed, probable and suspected cases kept increasing even with several interventions to contain the epidemic. The number of cases and deaths reported across all the eight previously affected countries (Guinea, Liberia, Mali, Sierra Leone, Nigeria, Senegal, Spain and the United States of America) were 28, 639 and 11,316 respectively [3], with an estimated case fatality rate (based on these figures) of 40%. Though EVD is deadly, measures such as washing of hands with soap and water, using hand sanitizers, and avoiding contacts with people who are infected with the condition and their body fluids can reduce cross-infection and help contain spread of the disease. This is because the virus is present in the bodily fluids of infected individuals and all items contaminated with such bodily fluids are potential sources of transmission [4–6].

During EVD outbreaks, health workers and family members who take care of EVD patients, mourners and undertakers who come into physical contact with the corpse as part of rites of passage (burial ceremonies) have a greater risk of infection through direct mode of transmission [6]. One can also get infected with EVD through indirect means such as handling contaminated clothes, materials and equipment. Therefore, individuals who handle or physically touch these items are also at high-risk of infection. As part of containment efforts, some of these high-risk practices including washing and handling of the corpse were banned in the past during EVD outbreaks [7,8]. Attending funerals have also been reported to facilitate transmission to distant areas. This is because funeral attendants get infected and carry the infection to their communities and this can turn small outbreak into a major epidemic [9].

Many studies have identified economic and sociocultural factors as key hindrances to timely identification of cases and implementing control efforts in the affected regions [10–12]. Adjusting such cultural practices in the wake of an EVD outbreak is therefore critical in controlling transmission and communities that have not transformed from these high risk practices have been reported to face challenges containing an outbreak [13].

In response to the West African EVD epidemic, the Government of Ghana established an inter-ministerial committee which was chaired by the Minister of Health to lead EVD preparation and containment plan. Through their leadership and collaboration with development partners, a national preparedness and response plan was developed in August 2014. The plan itemized Ghana’s preparation in five key areas: planning and coordination of all EVD-related activities; passive and active surveillance, situation monitoring and assessment across the country; management of suspected and confirmed cases; social mobilization and risk communication; and provision of logistics, security and financial resources [14]. In line with the EVD response plan, Ghana Health Service also intensified social mobilization and risk communication across the country. Besides, national, regional and district EVD response teams were constituted along the decentralized governance system in Ghana to implement the plan. Ghana Health Service also established EVD treatment centres across the country, organized training for frontline health workers and provided Personal Protective Equipment to health facilities [14]. Screening of international travellers at entry and exit points in Ghana was also started in Ghana.

Although Ghana did not record a confirmed EVD case, the country is at risk through cross-border travel of people from affected countries in West Africa (including EVD survivors), or through de novo animal-human transmission. This research was therefore designed to explore cultural and social practices that may influence the prevention and containment of EVD and how to address these factors in Ghana.

## Materials and Methods

### Ethics statement

The Institutional Review Board of Noguchi Memorial Institute for Medical Research (NMIMR) in Ghana reviewed and approved the proposal to conduct this study. The reference number for this protocol was NMIMR-IRB CPN 057/14-15. Informed written consent were solicited and obtained from all study participants before the study and data collected were anonymised.

### Study design

We adopted a qualitative study design and method of data collection. This strategy was employed because the purpose of our study was to gain deeper understanding of the relevant socio-cultural factors that may hinder EVD containment from both community members and health workers. We combined two qualitative study designs; phenomenology and narrative. Phenomenology approach to qualitative research is used to study participants’ perceptions, feelings, and lived experiences of a particular phenomenon in the community and how these knowledges can affect the person viewpoints concerning a specific situation [15]. Narrative research on the other hand allows study participants to describe their experiences in the community [16].

### Description of study area

We conducted this research in the Republic of Ghana which is located on West Africa's Gulf of Guinea. Ghana shares boundary with three neighbouring countries; Côte d'Ivoire, Togo and Burkina Faso to the west, east, and north respectively. Located at the southern border of Ghana is the Atlantic Ocean and the Gulf of Guinea. The population of Ghana is estimated as 24,658,823 with yearly growth rate of 2.4% [17]. The nation is divided into ten decentralized administrative regions. English is the official language of Ghana but there are about 75 local languages.

Despite the fact that we have 10 regions in Ghana, this study was conducted in five regions with a combined population of 15,764,171. These regions include: Western, Greater Accra, Volta, Ashanti and Northern. The two most populous regions in Ghana are the Greater Accra region (GAR) and Ashanti region (AR) with populations of 4,010,054 and 4,780,380 respectively. The population densities of Greater Accra region is 1,236 per square km whilst that of Ashanti region is 196 per square km [17]. The Volta (VR) and Western (WR) regions have a population of 2,118,255 and 2,376,021 respectively [17]. Furthermore, the Northern Region (NR) has a population of 2,479,461 [17].

**Fig 1:**
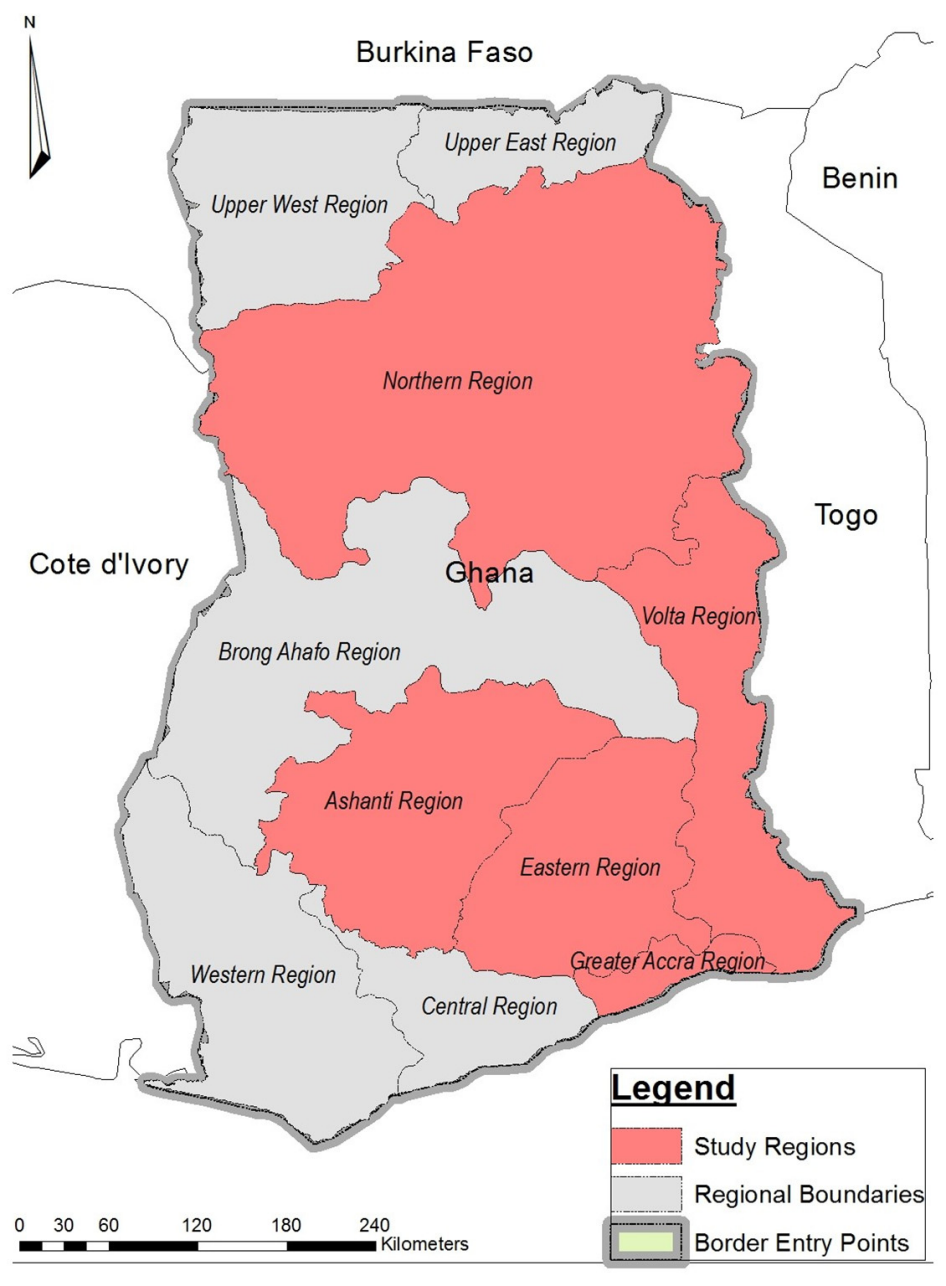
Map showing study areas and borders with neighbouring countries.

We considered population density, rural-urban factors and entry and exit points in Ghana as the criteria in selecting the five regions. Animal-to-human and human-to-human transmissions of EVD is faster in areas of high population density, therefore the two regions (GAR and AR) with high population densities were selected for the study [18]. The Greater Accra region which is the national capital of Ghana is also home to the main international airport and seaport. The Volta and Western regions share boundary with Togo and Cote D’ Ivoire respectively. The Western region also has seaport and therefore serves as point of entry for sea travellers. The Northern Region is the biggest region located in the northern Ghana and also shares boundary with neighboring Cote D’Ivoire. Fig 1 shows the regions that were selected for the study and their boundaries with neighbouring countries which serve as entry/exit points to/from Ghana.

### Data collection method

#### Focus group discussions

We conducted 25 focus group discussions (FGDs) in the entire study; 5 in each region (2 males, 2 females groups and 1 among nurses). To begin with, we stratified the selected region into urban and rural communities. After which we conducted two FGDs; 1 among males and 1 among female groups. Thus, 10 FGDs were conducted in rural areas and 10 in urban areas. In each region, an additional one FGDs was organized with mixed male and female nurses. In each FGD, 8–10 people were brought together for the discussion making a total of 235 respondents in the entire research. The discussions were moderated in ways that allowed every participant to make his/her contribution to all questions raised and it took between 60–90 minutes to complete one discussion.

#### In-depth interviews

In addition to the FGDs, we conducted 40 in-depth interviews (IDIs); 8 in each region covering rural and urban participants. The IDI participants were mainly community opinion leaders including chiefs, health workers, regional and district EVD response team members, and immigration officers at border towns. Two port health workers were also interviewed in regions with ports (Western, Ashanti and Greater Accra).

### Data collection

We designed semi-structured IDIs, and FGDs guides for the data collection. The guides were translated from English to the local language before the field work began. Back-to-back translation strategy was used to ensure that the versions in English were the same as those in the local languages. The FGDs and IDIs guides covered areas such as funeral practices, religious practices and other social activities in the community that can facilitate EVD transmission. We recruited 15 graduate research assistants, trained them after which they were deployed to the regions (3 to each region) to collect the data between 10^th^ February and 3^rd^ March, 2015.

### Selection of study participants

We included both males and females aged ≥ 18 years in line Ghana’s constitution for informed consent [19]. Purposive sampling was used to select participants who could provide in-depth information [20] on socio-cultural issues in the community. Research assistants entered each community through community leaders. The purpose of the study was explained to the community leaders who assisted the research assistants to select people from different sections of the community to constitute a group for the discussions. In selecting opinions leaders and chiefs, we took into consideration the ethnic diversity of the various communities. This was done to ensure that the data collected reflected views from various ethnic groups in the society. Members of EVD response teams were also purposively selected as they were supposed to lead preparation efforts in various regions and districts. Port health officials and immigration officers were also selected because they were trained to provide screening service at entry and exit points in Ghana. The participants were between 20–86 years and were either Christians, Moslems or adhered to African Traditional Religion. Some participants had no formal education whilst other had attained tertiary education. Whereas majority of the participants were married, a number of them were single.

### Data analysis

We sought permission from participants to record the FGDs and IDIs using digital voice recorders. We also took field notes which were turned into data document for analysis. The interviews that were conducted in local languages were translated to English by two independent language experts during transcription. The transcripts were again reviewed by listening to the voice recordings and comparing its content with the corresponding transcript. All the transcripts were imported into Nvivo 10 for windows for analysis.

We used framework method of qualitative data analysis in this study [21]. This method of data analysis involved the use of five iterative steps: familiarization, identifying a thematic framework, indexing, charting, mapping and interpretation. The actual data analysis process involves moving between these steps until the data analysis is completed [21]. The study team first read the transcripts to understand the key themes from the data which were used to develop a codebook to guide the data analysis. This codebook was discussed and accepted by the research team. Guided by this codebook, the data was then coded first as free nodes and later transformed to tree nodes.

We classified the data based on the source of the data and linked these sources to their attributes such as region, sex of respondent, and place of interview and nodes in Nvivo. We used the explore and query functions in Nvivo to provide a descriptive accounts of the data as required in framework analysis [22]. We then reviewed these descriptive accounts with existing literature to provide an explanation and interpretation of the data. Based on the data, we sketched frameworks which were grounded on the data to explain strategies to address socio-cultural practices that may hinder EVD containment in Ghana.

## Results

### Funeral practices and Ebola Virus Disease

In this study, it emerged that funerals are considered very important in all cultures in Ghana. As such celebrated with elaborate ceremonies and rituals from death to performing final funeral rites. These ceremonies and rituals vary between cultures and geographical areas across the country. Three main sub-themes emerged regarding ceremonies and rites related to funerals were identified. These include; care of the body of the deceased, burial practices and funeral practices. These subthemes and the common practices across the regions is shown in Table 1.

**Table 1:** High-risk funeral and burial practices in Ghana.

Region	Care of the corpse	Funeral practices	Burial practices
**Greater Accra**	Bathing the dead	Sharing cigarette and drinks with the dead	Holding the body of dead during burial
	Performing ablution	Hand Shaking	Burying deaths that occur at home without autopsy
**Western**	Bathing the dead	Use of handkerchief to wipe discharges from the dead	Holding the body of dead during burial
	Performing ablution	Widows rites	Burying deaths that occur at home without autopsy
		Drinking water used to rinse the dead	
		Sharing cigarette and drinks with the dead	
		Handshaking	
**Volta**	Bathing the dead	Use of handkerchief to wipe discharges from the dead	Holding the body of dead during burial
	Performing ablution	Handshaking	Burying deaths that occur at home without autopsy
**Ashanti**	Bathing the dead	Use of handkerchief to wipe discharges from the dead	Holding the body of dead during burial
	Performing ablution	Handshaking	Burying deaths that occur at home without autopsy
**Northern**	Bathing the dead	Use of handkerchief to wipe discharges from the dead	Holding the body of dead during burial
	Performing ablution	Throwing money at the dead which get contaminated by discharges from the dead	Burying deaths that occur at home without autopsy
		Handshaking	
		Bathing children with water used to rinse the body of the dead	
		Allowing children to drink the water used to rinse the body of the dead	

Regarding care of the body, the study found that in communities in Ghana, a person who is believed to have died a natural death has to be bathed at home. This practice (bathing) is considered an honour and respect for the dead by family members and community. Bathing is only forbidden in situations where the person is believed to have died from an unnatural cause such as suicide or any other unnatural causes depending on the community.

*“If you die a natural death in this community*, *we have to bath you before we start your funeral rites”*

(FGD, Male, Rural, WR)

*“…in [the] Ashanti culture*, *even if they bathed the body in the mortuary*, *when they bring it home they still bath it*, *so we have some old women around who bath the body”*

(FGD, Males, Urban, AR)

The study showed that bathing is often done without any form of protection except where the body is given to a funeral home for preparation towards burial. Any attempt to protect oneself at home during bathing is often viewed as a sign of disrespect for the dead. This practice was of great concern to members of the EVD response team and generally perceived as one of the cultural practices that may hinder prevention and control of EVD in Ghana as illustrated below:

*“Laughing*, *no*, *if you do that (protect yourself) it will mean you do not like the person who have died or is a sign of disrespect*. *No body wears any protective clothing*, *they use their bare hands”*

(FGD, Women, Rural, NR)

*“If someone dies and they take the person from the mortuary*, *they have to bath the corpse*, *so many people would be touching the body…this is big problem we need to overcome”*

(IDI, EVD Response Team Member, AR)

Filing pass the deceased body or staging the body on a palanquin was also identified as a common funeral practice in Ghana. In some instances, individuals in an attempt to show their extreme grief tend to lie on the body of the deceased during such funeral rites as indicated by this response in FGDs:

*“…*. *sometimes you see that the person sleeps on the body crying and others tell him to get up”*

(FGD, Male, Rural, AR)

“You find people lying on the body of the dead and touching some of the secretions from the body”

(FGD, Female, Urban, GAR)

Furthermore, some people use handkerchiefs and other materials to drive away flies that settle on the corpse. This occurs when the corpse is staged for long, in which case, it starts to decompose thereby attracting flies. Respondents also believed the dead also express their grief by weeping whilst being mourned. Therefore, wiping of the tears of the corpse was required in some instances as illustrated by respondents.

“There is usually one person who holds a handkerchief and drives away flies from the corpse”

(FGD, Male, Rural, AR)

“People believe that the dead also cry because water drips from the body and people clean their faces with handkerchief”

(FGD, Male, Rural, VR)

Closely related to his practice is throwing of money at the dead as part of funeral rites. These monies sometimes come into direct contact with bodily secretions should the corpse starts to decay. Such monies are often collected by the dirge singers and the undertakers as reward for their various roles in the funeral activities. Others also share cigarette or drinks with deceased to give opportunity to the dead to share in a habit he/she was engaged in whilst alive as illustrated:

*“Our funeral practices where we throw monies at the dead body as part of funeral rites is also risky if there should be Ebola in Ghana*. *Because*, *sometimes the corpse will decay and we throw such monies at the decomposed body…such monies can be contaminated”*

(FGD, Male, Urban, NR).

“…if someone dies some even share cigarette or drink with the dead to show solidarity/affection”

(FGD, Males, Urban, WR).

Widowhood rites also emerged as a high-risk funeral practice among southern Ghanaian. In this practices, the widow is made to drink the water used to rinse the dead husband to show her innocence in the death of the husband. This practice is often activated when there are suspicions that the widow could be responsible for the death of her husband.

“Some groups will also force the surviving spouse to drink part of the water used to bath the corpse to prove your innocence that you are not the cause of the death”

(FGD, Males, Urban, WR).

The study also showed that sometimes children are bathed with water used to rinse the body of dead as a way of fortifying children. This ritual is undertaken when the deceased is believed to possess special spiritual powers that should be transferred to grandchildren. Bathing this water is also believed to offer spiritual protection against evil spirits in the community especially among Northern Ghanaians.

*“In my tribe*, *when someone like a big man die*, *they use the water that has been used to rinse the dead to bath children*”

(FGD, Male, Rural, NR).

*“It is believed that it gives the children some spiritual power especially if this was a very powerful person in the community*”

(FGD, Male, Rural, NR)

The study generally revealed that majority of these high-risk funeral practices were part of the culture of the people and therefore very difficult to modify. However, some respondents in northern Ghana were of the view that if a particular death is believed to be due to Ebola, people may be unwilling to undertake such high-risk funeral and burial practices for fear of contracting the disease. Some also believe the use of some concoction could offer protection and prevent anybody who come in contact with the deceased’s body from getting infected as illustrated:

“*…*.*Actually*, *there will not be much problem if there is Ebola suspected case*. *If an Ebola person dies they will consider those alive first and would not want them to also be infected and because of that they will even prevent family members from touching the person*. *If they even decide to bath the body*, *they will take some precautions to avoid contracting the infections”*

(IDI, Opinion Leader, Male, NR)

*“…*.*If the person dies from a strange condition that you suspect you can get it by touching the body*, *we have some concoctions we prepare from herbs*. *You put your hand inside that preparation before touching the body and after touching and this will protect you”*

(FGD, Male, Rural, NR)

Nonetheless in the other regions, bathing the dead body was perceived to be mandatory and could therefore not be overlooked for fear of the reprisal effects from the dead. They were of the opinion that if the bathing is not carried out then the soul of the person does not go to rest in peace but will wander about and sometimes confront those who took the decision to deny the deceased this rite of passage:

*“We can’t stop*, *it’s a custom*, *because it’s usually the last respect you are giving*. *When you were born*, *you were bathed so when you die it should be done for you*, *there have been cases where dead bodies were not bathed and they come hunting [confronting] the family members”*

(FGD, Female, Rural, GAR)

### Daily praxis and practices and Ebola Virus Disease risk

Handshaking emerged as one of the everyday communal social norms that may influence Ebola prevention and containment. The study showed that handshaking are deeply embedded in all social gatherings such as funerals, religious congregations, and festivals. In fact, it is a daily occurrence when friends, work colleagues or acquaintance meet. To respondents, this was mandatory in some social settings and had deep roots in the culture and behavioral etiquettes of the people as illustrated by a respondent.

*“Shaking of hand s is very important in our society*. *If you refuse to accept handshakes you are seen as proud in this community*. *In funerals*, *you go round and shake everybody’s hand”*

(FGD, Female, Rural, AR).

*“…*.*Our religion has also made it that we shake hands after prayers which is also part of our culture”*

(FGD, Males, Urban, NR).

Another community practice that can promote the spread of Ebola in the community is the practices of sharing drinks in drinking bars. It is a common practice that one person would share his/her drink with another person using the same calabash. Closely related to this is the use of communal glasses by operators of drinking bars. Such glasses are often not washed well before they are used to serve another customer as illustrated by the following responses:

*“Those of us in the villages when we meet at palm wine joint*, *we inadvertently shake hands and sip drink from each other calabash”*

(FGD, Male, Urban, WR)

*“And taking of alcohol*, *someone drinks ‘‘akpeteshie” (local gin) from a cup then another person also comes and drinks from the same cup”*

(FGD, Male, Rural, GAR)

The study further revealed that during Eucharistic celebration by Christians, common cups are often used by members of the congregation. The communal use of cups is a high-risk practice for Ebola since the cups may come into contact with the saliva of users.

“In church…they pour wine into small cups and add bread and even if we are 1000 it’s the same 20 cups that is used for all so it can promote the spread of Ebola”

(FGD, Male, Rural, GAR).

Gender related social factors also emerged in both FGDs and IDIs across the country. These gender-related issues may increase vulnerability of either the male or female gender depending on whether the person belong to a matrilineal or a patrilineal descent. First and foremost, the study revealed that in all cultures, women were the prime caretakers with the responsibility of taking care of the sick at home and this included bathing, feeding and administering drugs. Hence, in the event that there should be an Ebola outbreak in Ghana, females will be more likely to get infected than their male counterparts. Men only offer to assist when the condition becomes critical which requires moving the sick person to another source of care:

*“We women do all the washing of clothes and all the items often used by a sick person*, *therefore if the person should have the condition*, *it means we will get it”*

(FGD, Female, Urban, VR).

*“Mostly the women take care of the sick in this community*. *However*, *when the condition becomes serious*, *then the men will called upon to help”*

(FGD, Female, Urban, NR).

In the Ashanti Region, and other Akan speaking regions in Ghana, inheritance is matrilineal and this also affects domestic care giving. This type of inheritance makes it obligatory for women to take care of sick people in their descent.

“As for us the Ashantis we inherit from the mother side so that is why the females have to look after the sick”

(FGD, Males, Urban, AR).

On the other hand, men in society are often in-charge of taking care of dead bodies. This also makes men more susceptible to Ebola should the death be as a result of EVD:

“Men are the people who often take care of dead bodies in our community”

(FGD, Female, Rural, WR).

Decision-making process also largely favoured men in the communities as men often make key decisions at the household level as showed by these responses:

*“There are homes where the woman has no say*, *what the man says is final”*

(FGD, Male, Rural, VR).

*‘You see the women are most of the times with the children so if they realize that any of the children is not well they (men) will tell us and we will ask them to take him or her to the hospital”* (FGD, Male, Rural, GAR).

### Health care seeking and Ebola Virus Disease risk behaviour

The study showed that some Ghanaians use multiple health care outlets in their communities including self-medication at home, buying drugs from license drugs store, and health facilities or a traditional healers. However, taking medications (both herbal and orthodox) at home or buying medicines from a licensed drug stores as first aid was reported to often precede reporting to any health facility. In FGDs, it was unanimously agreed that drugs are first purchased from the drug stores for treatment at home and if the sick person condition showed no improvement after some days, then the individual is sent to either the hospital or traditional healer. Others will take some herbal preparations first and then report to the hospital if their condition does not improve.

*“…If you have headache you buy medicine at the drug store and take but if you do not get better*, *then you quickly have to go to the hospital for care”*

(FGD, Female, Rural, AR)

*“If any of my families is not well*, *what I do is go to the drug store and buy drugs for the person*, *if after 3 days the person is not well then I will take the person to the hospital”*

(FGD, Female, Urban, GAR)

*“Truly*, *for this community*, *when a person is sick*, *you go to the bush to get some herbs to treat them*. *There are different types of herbs depending on the complain of the person*, *you give the herb that you know is capable of treating that condition but if this fails*, *you take them to the hospital”*

(FGD, Female, Rural, NR)

According to respondents, several factors determine the type of health care that is sought. Quick access to health care emerged as one of the factors that influence the place a sick person seeks health care.

*“I go to Abetiman hospital which is a private hospital and they attend to me quickly and because they know if they treat me well I will pay them and they will get their money but in the government hospitals when you get there the nurse will say*, *'wait for a while*, *we have not started work”*

(FGD, Female, Rural, AR)

Another consideration in seeking health care is the type of health condition. In the opinion of some community members, some conditions are not “sickness meant for the hospital”. These conditions are often described as spiritual or supernatural in origin and therefore unsuitable for hospital care. One of such conditions was mentioned as “yɔgu”, a Dagomba word in Northern Ghana which according to FGD discussants is characteristically similar to Ebola. The condition “yɔgu” translated to mean “deep forest” is a disease that people get from deep forest through either eating the touching an infected animal or eating the infected meat.

*“We have known a particular disease called “yɔgu” that once existed*. *It is similar to what you call Ebola*. *If one contracts it*, *they will die just us Ebola kills its victims so we are afraid of that disease because if even ten people touches a victim*, *they will all die*

(FGD, Male, Rural, NR).

*“Our elders have some herbal medicine that they use on victims of the “yɔgu” we talked about*. *When people touch them*, *they will not contract it*.*But if they don’t use the medicine on the victim*, *anyone who touches them will get the disease”*

(FGD, Male, Rural, NR)

### The influence of socio-cultural barriers to prevention

From the study most of the socio-cultural practices were perceived to be deeply rooted in tradition. Nonetheless, respondents were of the view that some of them could be modified in the event of Ebola outbreak through collaborations with chiefs and religious leaders in the community.

*“In my place here*, *everything lies on the chief so the nurses can tell the chief to tell his members about what not to do and what to do*. *If the chief announce it everybody will follow*. *If the chief should announce that henceforth*, *nobody should go round to shake hands at funeral again in their community*, *nobody will do that because you will fear to be sanctioned”*

(FGD, Female, Urban, AR).

*“…if the chief should give that directive to the men and they agree we will follow it but I don’t pray that the condition should get here because it kills so fast and in this community*, *it will spread very fast because of the way you live”*

(FGD, Female, Rural, NR)

People who adhere to the Islamic faith, generally believed that involving their religious leaders is the only practical ways of changing high-risk funeral and burial rites. Bathing the dead was believed to be part of their religious doctrine and could only be changed or modified by the Islamic leaders who understand the teachings of the Quran better.

*“When the Imams are involved*, *they know the Quran and what can be modified*. *I am sure if because of EVD we are told not to bath the dead again by the leader*, *we will obey*. *Because when some die they come to lead the prayer for the person”*

(FGD, Male, Urban, NR)

## Discussion

### Funeral and burial practices

Practice of bathing the body of the deceased is a major risk factor in EVD prevention and control as identified in this study. The people who bath the corpse are often not protected and several people may be involved in the process. This therefore puts all the people involved at risk of getting infected should it be a case of Ebola. In situations where bathing is performed by the undertakers of a particular clan, it implies that the infection could be transmitted to other clans in the community. Practices such as bathing the corpse, anointing people with water used to rinse the corpse, and staging the body for funeral rites and burial practices, are high-risk activities and have been reported to have exacerbated human-to-human transmission in Liberia and Sierra Leone [23]. In Guinea, 60% of all EVD cases had been linked to traditional burial practices [24]. In this study, almost all respondents acknowledged the high-risk inherent in these funeral and burial practices. Nevertheless, there was little readiness to change these practices as it would be seen as an affront to their culture. Despite that impression, with the fear and general perception of the high fatality of EVD among communities, it may be possible to induce a change if an EVD case should be confirmed in the nation. This however will require the involvement of community leaders coupled with close monitoring. Lesson learnt from the history of cholera in Ghana should guide this effort. It has been documented that two of the worst areas in Ghana that have been affected with cholera are Akplabanya and Nyanyano located in Ada and Winneba districts of Ghana respectively. These outbreaks were widely speculated to have resulted from the bringing in of relatives of Ghanaians who had died from cholera in Togo and Guinea for burial in these two fishing communities thereby infecting others [25]. In Sierra Leone, as many as 350 Ebola deaths were attributed to the funeral of a renowned traditional healer who got infected when treating EVD patient [24]. Involving community leaders’ especially religious leaders and strict monitoring will be required to ensure that people adhere to directives on safe funeral practices. Ghana should consider developing a safe funeral and burial practices documents which will consider inputs from various stakeholders. Lessons from Liberia has showed that funerary as well as caregiving practices could either be suspended or altered by community members when they are fully engaged [26]. Local community engagement is reported to be crucial in reducing transmission rates [27–29].

### Everyday communal practices and Ebola Virus Disease risk

The study showed that hand shaking is highly cherished in Ghanaian settings. The popularity of the handshake continue to grow and is seen as a sign of love, affection and concern in all social gatherings. Therefore, an individual stand the risk of being perceived to be rude where he/she refuses to accept a hand shake or offer one when it is required. Since, handshake is another medium of transmission of EVD, it will be important for information, education and communication messages to provide alternative greeting strategies that will limit the more physical contact inherent in the traditional handshakes. In a recent study, it was reported that the so-called high five and fist bump compared with a traditional handshake substantially reduce the transmission of infectious disease between individuals [30]. This should therefore be emphasized in health education strategies to help reduce physical contacts through handshakes. In the absence of alternatives, people are compelled to stick to the traditional ways of handshaking which increases the risk of transmission of infections such as EVD.

The use of communal cups and calabashes were mentioned as common and is a high risk social practice. This was not only reported as a common practice in drinking bars but also in the Eucharistic practice in churches. During the Eucharistic practice, common communion cups are used by the congregation. A classical study has showed that during the use of such communion glasses in church, several micro-organisms are deposited on the glasses [31]. This therefore requires serious attention in EVD prevention and control strategies. For the use of communal glasses and cups in drinking bars, EVD-related health education should highlight the potential risk in this practice. When people are sensitize to demand disposable cups, the bar operators will be compelled to make available disposable cups. In times of emergencies like EVD outbreak, a directive can also be given by the appropriate authorities to ban the use of communal cups and drinking glasses in bars. However, regarding the use of communion glasses, church leaders can be sensitize to stop the use of communal drinking glasses in their religious practices.

**Fig 2:**
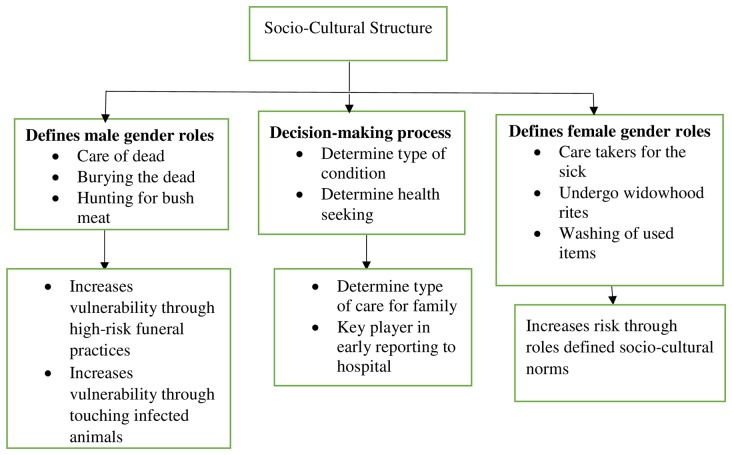
Socio-cultural practices and its influence on EVD-related risk.

The study showed that socio-cultural norms from society makes both men and women vulnerable to one form of high-risk behavior or the other. Socio-cultural norms require that females take care of sick at home and at the hospital. Taking care of the sick involves feeding and washing of the sick person. These activities may require coming into contact with bodily fluids of the sick person thereby increasing the risk of contracting the EVD. This social norm therefore increases the vulnerability of women than menfolk. Studies in the three epicenter countries (Liberia, Guinea and Sierra Leone) have revealed that EVD deaths is higher among women than men with 50.8% of the cases being women as of 7^th^ January, 2015 [32]. In Guinea, number of EVD cases among males and females were 1,309 and 1,410 respectively. Also in Sierra Leone, more females (4,151cases) were infected than males (3,891 cases) [32]. Similarly, in previous ebola outbreaks in Congo, Gabon and Uganda, women were reported to more affected [32]. In the same vein, the study showed that some socio-cultural norm also make males responsible for certain activities in the community which are considered high-risk activities. Though some women may be called upon to care for the dead, this activity is mostly undertaken by men hence, if the deceased was a case of EVD, it will increase the risk of men getting infected. The study further showed that decision making tend to favour men as they are responsible for making important decisions such as determining the health seeking behaviour of their household. It is therefore important for such gender related issues to be considered in any preparation towards containing EVD. Fig 2 shows how socio-cultural practices can increase an individual’s risk of infection.

### Health seeking behaviour and Ebola Virus Disease

The study showed that multiple treatment outlets are often sought either simultaneously or in sequence. From all indications, the first point of call for a sick person is not the hospital or herbalist/ritualist but care at home through the use of either drugs obtained from drugstores, or passed on from other relatives, with some people taking herbal preparations. An earlier study in Nigeria reports that about a quarter (26%) of the study population believed local and traditional medicines (herbs and concoctions) could provide a cure for EVD [33]. Given the nature of EVD, this approach will be an impediment in efforts to contain it should there be an outbreak. This is because sick people will be kept at home and only sent to hospital or herbalist/ritualist after their conditions have become worse and/or they have infected other members of their household. Since these first aid treatments are sometimes bought from drug stores in the community, in the event of an Ebola outbreak in Ghana, involving caretakers of drug stores may be essential since they are the first point of call for sick people in the community. They should be equipped with the knowledge and skills required to screen people for EVD so that they can screen their customers appropriately. The training of frontline health workers should therefore extend to members of chemical license sellers associations in communities because of their potential role in identifying cases of EVD.

Nonetheless, majority of respondents in the study stated that if a sick person is suspected to be carrying EVD, the hospital will be the first point of call for health care. Despite this, the mere fact that signs and symptoms of EVD are akin to endemic conditions like malaria, diarrhea, typhoid fever (enteric fever) and pneumonia, means that people might misconstrue the early clinical presentation of EVD for these conditions. Therefore, home care will still persist until such a time that haemorrhagic signs manifest as this appears to be the only definite indication that suggests an EVD disease to community members. Haemorrhagic manifestation are later signs of EVD and do not occur immediately [4,5], therefore the possibility of infecting household members and close associates before health care is sought at a biomedical health facility could be high.

**Fig 3:**
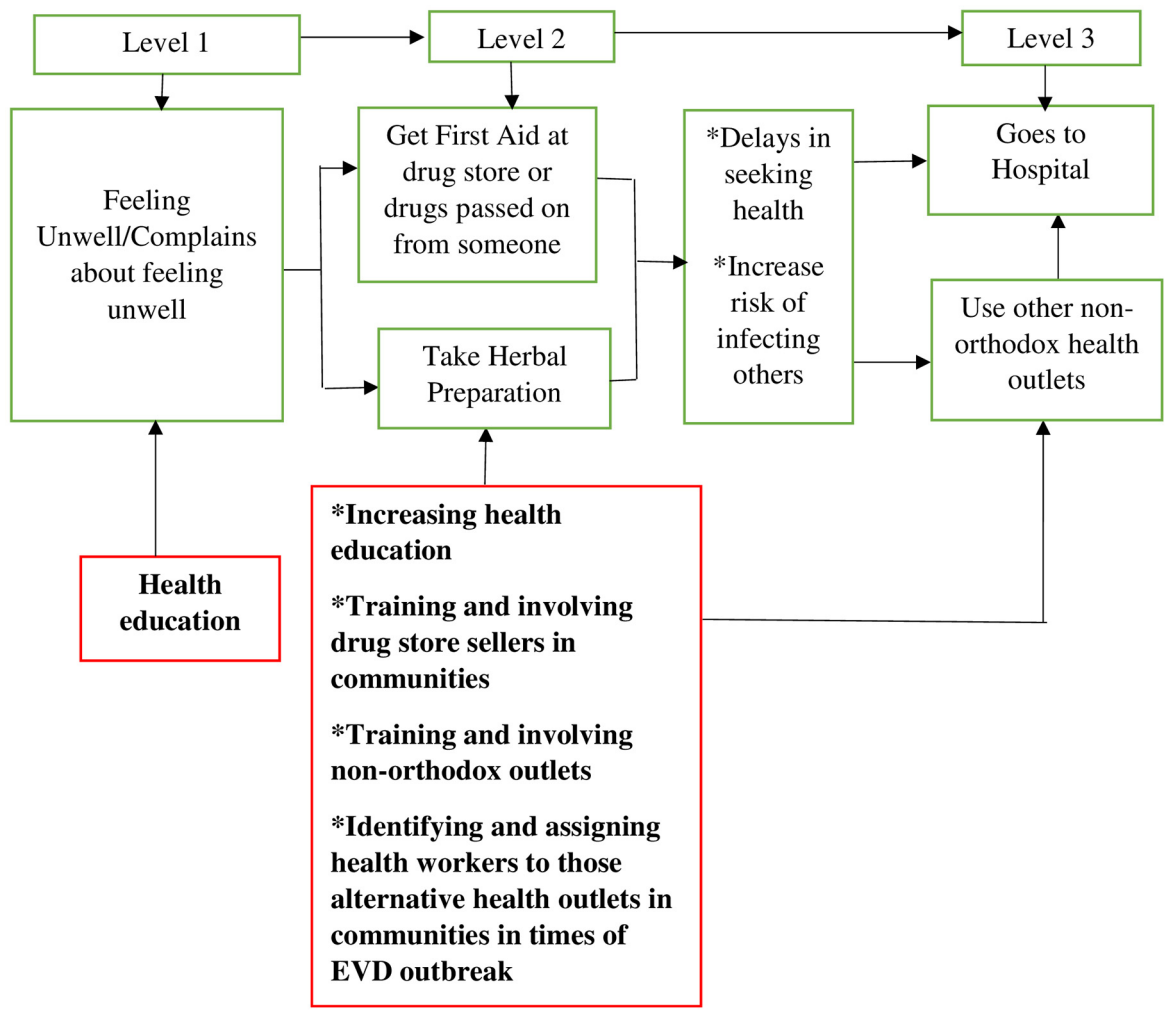
Diagrammatic representation of health seeking behaviour and strategies to contain EVD during an outbreak.

The summary of the health seeking behavior of Ghanaians and the ways to address delays are highlighted on Fig 3. Involving the various health outlets in EVD prevention and control will ensure that people that report to such outlets are screened appropriately to avoid delay which will invariably increase the risk of infecting household and community members.

### Methodological considerations

Interviews conducted in local language were translated to English by independent language experts and these translation were verified by another language expert. Nevertheless, there is still the possibility of words losing their original meaning during the translation procedure. To circumvent this weakness, we placed emphasis on well-entrenched themes during coding rather than the specific phrases and word used by participants. Where it was impossible to find a suitable English word or phrase for a local term, we maintained the local word and placed the direct translation in English in parenthesis. We were also only able to conduct the study in five out of the ten regions in Ghana. This therefore has implications in generalizing the findings of the study to the regions that were not covered in the study. Nonetheless, we ensured that regions that were selected fairly represented the main ethnic groups in the country.

### Conclusions

The study showed across Ghana, high risk socio-cultural practices were common and generally perceived to have deep root in culture, therefore making such practices indispensable. Socio-cultural factors may hinder containment efforts in the event of an outbreak. Community leaders should be engaged in any social mobilization to modify these practices as part of preparation efforts. Social mobilization through community leaders and culturally appropriate health education are required.
